# Risk Effects of rs1799945 Polymorphism of the *HFE* Gene and Intergenic Interactions of GWAS-Significant Loci for Arterial Hypertension in the Caucasian Population of Central Russia

**DOI:** 10.3390/ijms24098309

**Published:** 2023-05-05

**Authors:** Tatiana Ivanova, Maria Churnosova, Maria Abramova, Irina Ponomarenko, Evgeny Reshetnikov, Inna Aristova, Inna Sorokina, Mikhail Churnosov

**Affiliations:** Department of Medical Biological Disciplines, Belgorod State National Research University, 308015 Belgorod, Russia; 4602@bsu.edu.ru (T.I.); churnosovamary@gmail.com (M.C.); abramova_myu@bsu.edu.ru (M.A.); ponomarenko_i@bsu.edu.ru (I.P.); reshetnikov@bsu.edu.ru (E.R.); aristova@bsu.edu.ru (I.A.); sorokina@bsu.edu.ru (I.S.)

**Keywords:** hypertension/blood pressure genes, arterial hypertension, SNP, association

## Abstract

The aim of this case-control replicative study was to investigate the link between GWAS-impact for arterial hypertension (AH) and/or blood pressure (BP) gene polymorphisms and AH risk in Russian subjects (Caucasian population of Central Russia). AH (n = 939) and control (n = 466) cohorts were examined for ten GWAS AH/BP risk loci. The genotypes/alleles of these SNP and their combinations (SNP–SNP interactions) were tested for their association with the AH development using a logistic regression statistical procedure. The genotype GG of the SNP rs1799945 (C/G) HFE was strongly linked with an increased AH risk (ORrecGG = 2.53; 95%CIrecGG1.03–6.23; ppermGG = 0.045). The seven SNPs such as rs1173771 (G/A) *AC026703.1*, rs1799945 (C/G) *HFE*, rs805303 (G/A) *BAG6*, rs932764 (A/G) *PLCE1*, rs4387287 (C/A) *OBFC1*, rs7302981 (G/A) *CERS5*, rs167479 (T/G) *RGL3*, out of ten regarded loci, were related with AH within eight SNP–SNP interaction models (<0.001 ≤ pperm-interaction ≤ 0.047). Three polymorphisms such as rs8068318 (T/C) *TBX2*, rs633185 (C/G) *ARHGAP42*, and rs2681472 (A/G) *ATP2B1* were not linked with AH. The pairwise rs805303 (G/A) *BAG6*–rs7302981 (G/A) *CERS5* combination was a priority in determining the susceptibility to AH (included in six out of eight SNP–SNP interaction models [75%] and described 0.82% AH entropy). AH-associated variants are conjecturally functional for 101 genes involved in processes related to the immune system (major histocompatibility complex protein, processing/presentation of antigens, immune system process regulation, etc.). In conclusion, the rs1799945 polymorphism of the *HFE* gene and intergenic interactions of *BAG6*, *CERS5*, *AC026703.1*, *HFE*, *PLCE1*, *OBFC1*, *RGL3* have been linked with AH risky in the Caucasian population of Central Russia.

## 1. Introduction

Arterial (essential) hypertension (AH) is known as a disorder with high blood pressure (BP) [1]. AH is one of the most frequent diseases in the world—in 2015, AH was registered in 1.13 billion people [2]. AH appeared in 30–45% adults, with a predominant prevalence (>60%) in individuals aged >60 years [3]. AH is a serious risk factor for the manifestation of coronary heart disease, stroke, chronic kidney disease and dementia [4]. The risk of coronary heart disease and stroke doubles with an increase in systolic BP (SBP) for every 20 mmHg (starting from 115 mmHg) and diastolic BP (DBP) for every 10 mm Hg (starting from 75 mm Hg) [5]. The largest quantity of SBP-related deaths is caused by coronary heart disease, hemorrhagic and ischemic stroke [6].

The role of hereditary determinants in the formation of BP indicators is highly important and is beyond doubt [7,8,9,10,11,12,13]. The materials gained in the twin/family studies reveal a weighty heredity influence on the BP level, which according to different authors, varies on average within 30–55% [12]. In accordance with the catalog of genome-wide studies, GWAS (https://www.ebi.ac.uk/gwas/search?query=hypertension (accessed on 10 September 2022)), there is information on 118 performed GWAS, as a result of which 586 polymorphisms associated with AH were identified (the information is relevant at the end of 2022). If we also take into account the data obtained in full-exome studies (EAWAS), the number of AH-involved polymorphic loci will exceed 1000 [10]. According to Padmanabhan et al. (2021), at the moment, there is publication information of about 1.5 thousand GWAS SNPs linked with various BP phenotypes (systolic/diastolic/mean/pulse BP) [13]. Considering the accessible data on “ordinary” associative studies of AH from the positions of diverse candidate genes [14,15,16,17,18,19,20,21,22,23], the amount of AH-associated polymorphisms can reach several thousand.

With this mega-large-scale amount of genetic information available to research teams dealing with the problem of the hereditary nature of AH, it is very problematic to give an answer to the question, “what are the specific polymorphisms/genes from the currently known more than several thousand such polymorphisms/genes (of which more than 1.5 thousand GWAS/EAWAS-significant), determine the susceptibility to AH in the population of this region (including residents of Central Russia)?”, since not everyone, even GWAS/EAWAS-significant polymorphism, will determine the susceptibility to AH in the study population. In this regard, replication studies are becoming particularly relevant [24,25,26,27,28], aimed at confirming (or, conversely, refuting) the role of GWAS gene polymorphism in the AH formation in residents of a particular territory having their own characteristics of the genetic “constitution”, the action of environmental factors, intergenic and gene–environmental relationships, etc., predetermining and features of the candidate genes involvement in the disease (AH) formation.

This case-control replication study estimated AH risk linked with GWAS impact loci in genes correlated with AH/BP in Caucasian populations of Central Russia.

## 2. Results

The distribution of genotypes in both AH (*p* ≥ 0.098) and control (*p* ≥ 0.251) groups were in compliance with H-We (Table S1). The genotype GG of the SNP rs1799945 (C/G) *HFE* was strongly linked with an increased AH risk in both Model 1 (OR_recGG_ = 2.53; 95%CI_recGG_1.03–6.23; p_recGG_=0.043; p_permGG_ = 0.045; power = 89.65%) and Model 2 (OR_recGG_ = 2.48; 95%CI_recGG_1.02–6.07; p_recGG_ = 0.045; p_permGG_ = 0.046; power = 88.37%) (Table 1). The high identical results on the relationship of rs1799945 (C/G) *HFE* with AH, obtained by us in both Model 1 (covariates list included in the analysis were the following: BMI; TG; TC; HDL-C; LDL-C; blood glucose; smokers) and Model 2 (in addition to the covariates of Model 1, two more factors such as low physical activity and consumption of fatty food with a high fat content were included in the analysis as confounders), may be due to the fact that the AH-impact phenotypic effects of these two additional covariates included in Model 2 have already been taken into account in the AH-significant phenotypic effects of the covariates of Model 1. Like this, BMI and lipid status indicators (TC; TG; LDL-C; HDL-C) (covariates of Model 1) are strongly correlated with the level of fatty food intake (additional covariate of Model 2), and the BMI parameter (covariate of Model 1) will be significantly linked with the level of physical activity (additional covariate of Model 2). These results allow us to use only a list of Model 1 covariates when studying the intergenic interactions that determine susceptibility to AH (the next stage of our study).

Alongside this, seven SNPs, such as rs1173771 (G/A) *AC026703.1*, rs1799945 (C/G) *HFE*,rs805303 (G/A) *BAG6*, rs932764 (A/G) *PLCE1*, rs4387287 (C/A) *OBFC1*, rs7302981 (G/A) *CERS5*, rs167479 (T/G) *RGL3*, out of ten regarded loci were related with AH within eight SNP–SNP interaction models (two-(p_perm-interaction_ ≤ 0.047), three-(p_perm-interaction_ ≤ 0.006), and four-(p_perm-interaction_ ≤ 0.001) level (SNP) models were considered, and two, three and three models, respectively, were significant) (Table 2). Three polymorphisms such as rs8068318 (T/C) *TBX2*, rs633185 (C/G) *ARHGAP42*, and rs2681472 (A/G) *ATP2B1* were not linked with AH.

**Figure 1:**
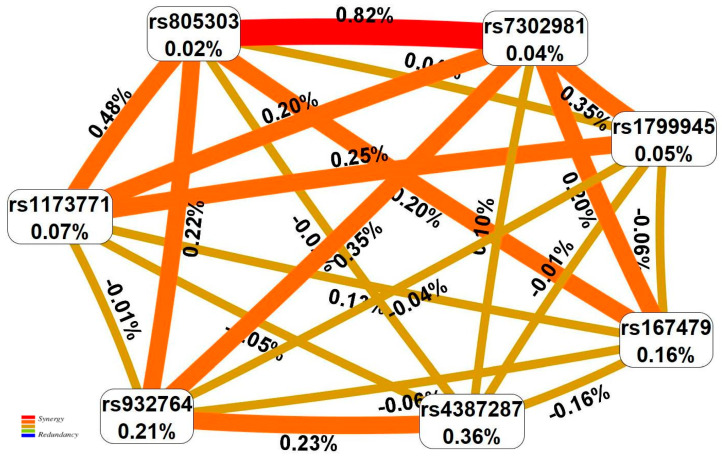
The entropy graph of the SNP × SNP interactions with AH based on the MDR analysis. Positive values of entropy indicate synergistic interactions, while the negative values indicate redundancy. The red and orange colors denote strong and moderate synergism, respectively, brown color denotes the independent effect, and green and blue colors denote moderate and strong antagonism.

Importantly, firstly, two loci such as rs805303 (G/A) *BAG6* and rs7302981 (G/A) *CERS5* were engaged in the maximum number (eight (100%) and six (75%), respectively) of AH-involved intergenic interactions models (Table 2). The pairwise SNP–SNP combination between rs805303 (G/A) *BAG6* and rs7302981 (G/A) *CERS5* has been priority in determining the susceptibility to AH (included in six out eight SNP–SNP interaction models (75%) (Table 2) and described 0.82% AH entropy (Figure 1)) in comparison with both individual loci (only 0.02–0.36% of entropy is determined (Figure 1)) and other loci interactions (defining less than 0.48% of entropy) (Figure 1).

Secondly, four-SNP models such as rs7302981 (G/A) *CERS5* × rs805303 (G/A) *BAG6* × rs1173771 (G/A) *AC026703.1* × rs167479 (T/G) *RGL3* have the highest Wald indicators (47.36, p_perm-interaction_ < 0.001), which “show” its leading value in predisposition to AH (Table 2). Thirdly, thirty-six AH-involved genotype combinations were modeled (Table S2), among which the largest effect (the biggest values (+/−) of the regression coefficient *beta*) has several up-susceptibility and down-susceptibility combinations such as rs7302981-AA *CERS5* × rs805303-GG *BAG6* × rs1173771-AA *AC026703.1* (*beta* = 2.34 *p* = 0.034), rs7302981-GA *CERS5* × rs805303-GG *BAG6*×rs1173771-AA *AC026703.1* (*beta* = 2.38 *p* = 0.022) and rs7302981-GG *CERS5* × rs805303-AA *BAG6* × rs1173771-GG *AC026703.1* × rs167479-GG *RGL3* (*beta* = −2.70 *p* = 0.021), rs7302981-GG *CERS5* × rs1799945-CC *HFE* × rs805303-GG *BAG6* × rs167479-GG *RGL3* (*beta* = −2.15 *p* = 0.050).

### 2.1. Functional Annotation of AH-Associated SNPs

#### 2.1.1. Non-Synonymous and Epigenetic-Significant Loci

There have been four missense SNPs presented (rs7302981 (G/A), rs1799945 (C/G), rs167479 (T/G) and rs1046089 (G/A)) among 88 examined polymorphic genetic variants (7 AH-associated loci and 81 linked with them) leading to amino acid (AA) permutations (Cysteine9Arginine, Histidine63Aspartic acid, Proline162Histidine and Arginine1740Histidine respectively) in 4 AH-causal proteins (CERS5, HFE, RGL3 and PRRC2A) (Table S3). These AA substitutions are characterized by SIFT “deleterious” (Proline162Histidine, Arginine1740Histidine), “tolerated” (Cysteine9Arginine, Histidine63Aspartic acid) predictive parameters, “probably damaging” (Histidine63Aspartic acid, Proline162Histidine, Arginine1740Histidine), and “benign” (Cysteine9Arginine) PolyPhen predictive parameters (Table S3).

Among eighty-eight possible AH loci, 11.36% of SNPs (n = 10) were located in conservative nucleotide DNA sequences, 56.81% (n = 50) were placed in introns, 5.68% (n = 5) were localized in five gene exons such as *COX14/LASS5*, *PRRC2A*, *HFE*, *RGL* (non-synonymous variants), *GPD1* (synonymous variant), and 4.55% (n = 4) were disposed in 3′-UTR (*COX14/LASS5*) and 5′-UTR (*OBFC1*) gene regions (Table S4). The bioinformatics data depict the presence of analyzed loci in the places of the supposed enhancers (n = 39; 44.32%) and promoters (n = 17; 19.32%), DNA-ase hypersensitive sites (DHsites) (n = 31; 32.33%), the probable areas of binding to transcription factors (TrF) (n = 74; 84.91%) and regulatory proteins (n = 16; 18.18%) (Table S4). In general, 88 possible polymorphic AH loci can potentially participate in the epigenetic regulation of the activity of 22 adjacent genes such as *RP11-411N4.1, RGL3, PRRC2A, PLCE1, OBFC1, HFE, NPR3, GPD1, LY6G5B, GPD1, LIMA1, CSNK2B, HIST1H1T*, *COX14, HIST1H4C, CERS5, HIST1H2BC, AC026703.1, LASS5, C12orf62, BAG6, HIST1H2AC*) (Table S4). A weighty number of AH-involved loci exhibit their epigenetic effects in AH target organs, such as the adult/fetal heart, aorta. For instance, in the conjectural promoter/enhancer gene regions in the fetal heart, there are loci rs4387287 (C/A) *OBFC1* and rs7302981 (G/A) *CERS5* in the right atrium, for left ventricles and aorta, rs4387287 (C/A) *OBFC1*, and for the right ventricle, rs1173771 (G/A) *AC026703.1*, rs805303 (G/A) *BAG6,* rs4387287 (C/A) *OBFC1*, rs7302981 (G/A) *CERS5.*

The largest number of strongly coupled loci (n = 34), including polymorphisms with any regulatory effects (n = 32), has been registered for our locus rs7302981 (G/A) *CERS5* (Table S4). Two of these loci such as rs78594839 and rs10538142 were located in the DNA regions that interacted with the largest number of TrF (11 each) such as Cart1, Dbx1, Evi-1, Foxp1, HDAC2, HNF1, Hoxa10, Hoxa5, Hoxb13, Sox, Zfp105 and Fox, Foxa, Foxd3, Foxf1, Foxi1, Foxj1, Foxj2, Foxp1, Foxq1, Sox, Zfp105. Three-fourths of all polymorphic loci strongly linked to rs7302981 (G/A) *CERS5* (26 of 34, 76.47%) were functionally significant (located in the DNA positions of the presumable promoters/enhancers/DHsites) in the AH target organs (adult/fetal heart, aorta). According to the information on chromatin states (HaploReg data based on 25-state model using 12 imputed marks), several polymorphic loci such as rs836179, rs836180, rs7967705 have the strongly pronounced epigenetic impact in all examined AH target organs (fetal heart, right atrium, left and right ventricles, aorta of adult).

#### 2.1.2. Plausible Gene Expression (eQTL) and Splicing (sQTL) Regulatory Potential of AH-Involved SNPs

The four AH-associated loci, such as rs7302981, rs4387287, rs805303, rs1799945, independently and due to strongly linked loci (33 SNPs out of 81 strongly linked loci were *cis-* and *trans-*eQTL significant, 40.74%) determine the expression of nine genes in human blood (peripheral) (*LY6G5C, AIF1, LIMA1, HCP5, SLK, HSPA1B, LASS5, TRIM38,* and *ALAS2*) (blood eQTL browser materials are presented in Tables S5 and S6).

At that, strongly pronounced gene expression was found with regulatory potential of all 7 AH-correlated loci and 74 coupled SNP (91.36% among all 81 examined LD SNP) in relation to 78 genes in other various organs (*ABHD16A, AIF1*, *APOM*, *AQP5*, *ASIC1*, *ATF1*, *ATF6B*, *ATP6V1G2*, *BAG6*, *BTN2A3P*, *C4A*, *C4B*, *C6orf48*, *CCHCR1*, *CERS5*, *CLIC1*, *COX14*, *CSNK2B*, *CTC-510F12.3*, *CYP21A1P*, *CYP21A2*, *DDAH2*, *DIP2B*, *DXO*, *GPD1*, *ANK1*, *GSTO1*, *GUSBP2*, *HCG22*, *HCP5*, *HDAC1P1*, *HFE*, *HIST1H3E*, *HLA-B*, *HLA-DQB1*, *HLA-DRB5*, *HLA-DRB6*, *HLA-S*, *HSPA1A*, *LARP4*, *LIMA1*, *LY6G5BLY6G5C*, *LY6G6C*, *LY6G6D*, *MICB*, *LY6G6E*, *LY6G6F*, *MIR6891, MPIG6B, NCR3, NEU1, NPR3, POU5F1, PRRC2A*, *RGL3*, *RNF5*, *RP11-457M11.5*, *RP11-541N10.3*, *RP3-405J10.3*, *RP4-605O3.4, SH3PXD2A, SH3PXD2A-AS1, SLC17A1, SLC17A3, SLK, SMARCD1, STK19, STK19B, STN1, TRIM38, U91328.19, UQCRHP1, VWA7, WASF5P, XXbac-BPG248L24.12, ZBTB12, ZNF322*) (GTExproject information is showed in Tables S7 and S8). It is important to highlight the serious capability to eQTL regulation of considered polymorphisms, as in the AH target organs such as arterial vessels (*VWA7*, *HFE*, *ABHD16A*, *LY6G5B*, *CYP21A1P*, *BAG6*, *ATF1*, *HLA-DRB5*, *SMARCD1*, *LY6G5C*, *C4A*, *COX14*, *RP4-605O3.4*, *RP4-605O3.4*, *LY6G5B*) and the heart (*CSNK2B*, *C4A*, *HLA-DRB5*, *CERS5*, *CYP21A1P*, *HIST1H3E*, *DDAH2*, *RP4-605O3.4*, *LY6G5B*, *STN1*), as well as in organs involved in the AH biology such as various parts of the brain (cortex; basal ganglia; hypothalamus; pituitary gland; black substance, etc.) (*ABHD16A*, *CERS5*, *CYP21A1P*, *DDAH2*, *HIST1H3E*, *LY6G5B*, *LY6G5C*, *MPIG6B*, *RP4-605O3.4*, *SH3PXD2A*), adrenal gland (*NPR3*, *SLC17A1*, *SLC17A3*, *GUSBP2*, *DDAH2*, *LY6G5B*, *ABHD16A*, *RP4-605O3.4*, *COX14*), thyroid (*HFE*, *TRIM38*, *LY6G5C*, *LY6G5B*, *HLA-DRB5*, *CCHCR1*, *CYP21A1P*, *C4A*, *RNF5*, *ABHD16A*, *LY6G6F*, *RP4-605O3.4*, *CERS5*, *LIMA1*, *COX14*, *ASIC1*), adipose (*ABHD16A*, *ASIC1*, *BTN2A3P*, *C4A*, *C6orf48*, *CCHCR1*, *CERS5*, *COX14*, *CYP21A1P*, *CYP21A2*, *DDAH2*, *HFE*, *HLA-B*, *HLA-DRB5*, *LY6G5B*, *LY6G5C*, *RP11-541N10.3*, *RP4-605O3.4*, *STK19B*, *STN1*, *U91328.19*, *VWA7*), skeletal muscle (*HIST1H3E*, *LY6G5C*, *LY6G5B*, *DDAH2*, *GPANK1*, *HLA-DRB5*, *ATF6B*, *APOM*, *C4A*, *RNF5*, *CSNK2B*, *PRRC2A*, *CYP21A1P*, *CLIC1*, *STN1*, *RP4-605O3.4*, *COX14*, *ATF1*, *DIP2B*), blood (*HIST1H3E*, *LY6G5C*, *LY6G5B*, *HLA-DRB5*, *C4B*, *PRRC2A*, *CYP21A2*, *VWA7*, *C4A*, *CYP21A1P*, *AIF1*, *C6orf48*, *RP4-605O3.4*, *ATF1*, *LIMA1*), etc. (Tables S7 and S8).

We observed the connection of the genetic polymorphisms under consideration (7 AH causal loci/65 SNP in LD) with the intron splicing regulation of 32 genes (*PRRC2A*, *AIF1*, *ATF6B*, *BAG6*, *C6orf48*, *CCHCR1*, *CYP21A1P*, *CYP21A2*, *DDX39B*, *FLOT1*, *GPANK1*, *LSM2*, *HLA-DQA1*, *HLA-DRB1*, *HLA-DRB5*, *HLA-DRB6*, *LST1*, *LY6G5B*, *LY6G5C*, *LY6G6C*, *MICA*, *STK19*, *STK19B*, *VARS*, *ATF1*, *CERS5*, *COX14*, *FAM186A*, *RP4-605O3.4*, *HFE*, *SMARCD1*, *SH3PXD2A-AS1*) including in disease target organs such as arterial vessels (aorta, coronary artery, etc.) (*BAG6*, *HLA-DRB1*, *HLA-DRB5*, *HLA-DRB6LSM2*, *STK19B*, *GPANK1*, *ATF6B*), heart (*GPANK1*, *HLA-DRB1*, *HLA-DRB5*, *HLA-DRB6*, *STK19B*, *LY6G5C*) and organs significant for AH pathogenesis: brain (cortex; basal ganglia; pituitary) (*LY6G5C*, *BAG6*), adrenal gland (*CCHCR1*, *BAG6*, *CYP21A2*, *CYP21A1P*), thyroid (*GPANK1*, *HLA-DRB1*, *STK19*, *HLA-DRB5*, *CCHCR1*, *HLA-DRB6*, *STK19B*, *BAG6*, *FLOT1*), adipose (*HLA-DRB6*, *BAG6*, *HLA-DRB1*, *AIF1*, *HLA-DRB5*), skeletal muscle (*GPANK1*, *HLA-DRB5*, *BAG6*, *HLA-DRB6*, *HLA-DRB1*, *HLA-DRB5*, *CCHCR1*), and blood (*HLA-DRB5*, *BAG6*, *HLA-DRB6*, *AIF1*, *GPANK1*, *HLA-DRB1*, *LY6G5C*) (Tables S9 and S10).

#### 2.1.3. Pathway Analysis of AH-Associated Genes

Based on the 101-gene (previously, these genes functionally related to 88 disorder-involved polymorphic genetic variants (7 AH-associated/81 linked with them) were identified) enrichment analysis results, an extremely large number (about 150) of biological pathways was discovered (Table S11). Among the pathways, processes related to the immune system prevailed; the greatest statistical significance was shown by such pathways as major histocompatibility complex (MHC) protein (PC00149, p_fdr_ = 4.18 × 10^−11^), antigen processing and presentation (GO:0019882, p_fdr_ = 4.74 × 10^−10^), positive regulation of immune system process (GO:0002684, p_fdr_ = 3.66 × 10^−7^), adaptive immune response based on somatic recombination of immune receptors built from immunoglobulin superfamily domains (GO:0002460, p_fdr_ = 2.46 × 10^−6^), lymphocyte mediated immunity (GO:0002449, p_fdr_ = 2.73 × 10^−6^), etc.

**Figure 2:**
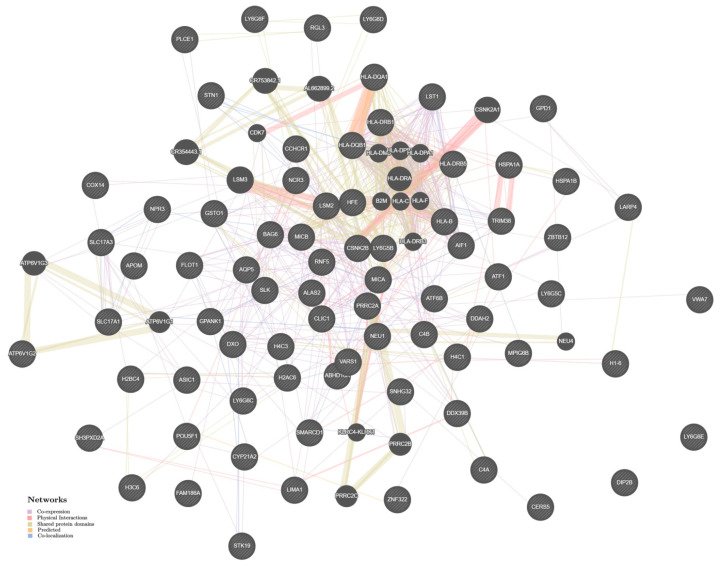
The interaction networks of the candidate genes for the AH in various tissues/organs inferred using GeneMANIA (http://genemania.org (accessed on 16 October 2022)). The candidate genes are cross-shaded.

The simulated network of intergenic interactions at predisposition to AH (Figure 2) is based on the following hypothetical “mechanisms”: co-expression (48.87%), physical interactions (30.06%), common protein domains (7.78%), joint localization (7.56%), and forecast interactions (5.72%). Among the “main” interacting genes “additionally” included in the genetic network (besides the 101 AH-recognized genes), the first rank positions are occupied by the two genes *LSM3* and *CSNK2A1*. The most pronounced interactions (the physical interactions weight index is 1) were discovered for such gene pairs as *HLA-DQB1/HLA-DQA1*, *LSM3/LSM2*, *CSNK2A1/CSNK2B* (Table S12).

**Figure 3:**
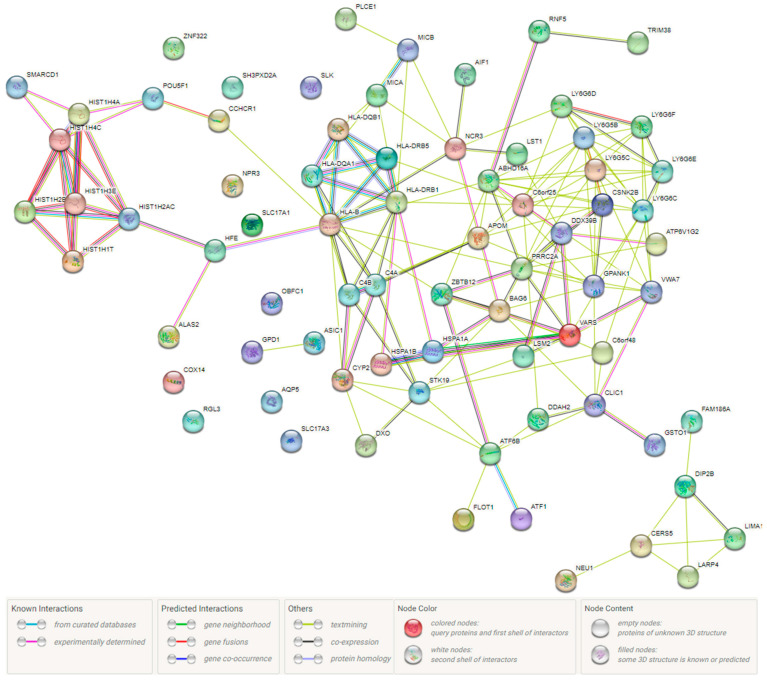
AH-involved protein–protein interaction networks inferred using STRING resource.

When investigating AH-involved protein–protein interaction (PPI) networks and functional enrichment analysis performed using the STRING online resource, the following data were obtained. Figure 3 shows that the AH-involved proteins significantly interact with each other (PPI enrichment *p* value < 1.0 × 10^−16^) and such PPI as Ly-6 antigen/uPA receptor-like/acetylcholine receptor regulator activity (p_FDR_ = 3.16 × 10^−16^), Tenascin-X/Proline-rich protein 3 (p_FDR_ = 6.23 × 10^−8^) and proline-rich protein 3/TRIM10/RING-HC finger (p_FDR -_ = 6.23 × 10^−8^) are of paramount importance in these processes. These PPI are carried out on the basis of protein domains such asimmunoglobulin C1-set domain (p_FDR_ = 9.59 × 10^−8^), Class I histocompatibility antigen, domains alpha 1 and 2 (p_FDR_ = 0.003) and Class II histocompatibility antigen, beta domain (p_FDR -_ = 0.013).

**Figure 4:**
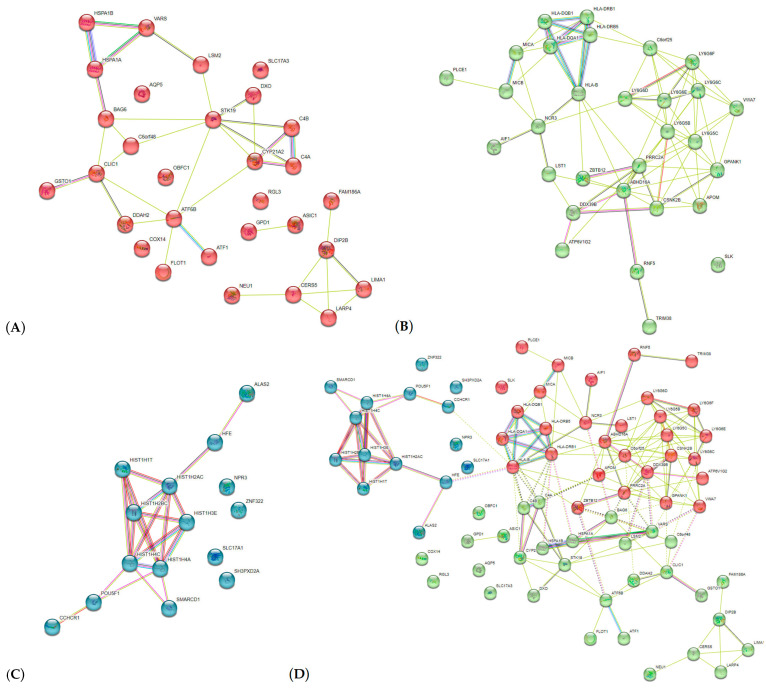
AH-involved protein–protein interaction clusters inferred using STRING resource (three groups of PPI clusters are highlighted in color: cluster 1, red (**A**); cluster 2, green (**B**); cluster 3, blue (**C**); summary of three clusters (**D**)).

Among the PPI, three clusters of functionally related proteins can be distinguished (Figure 4D, clusters are indicated by different colors, red, green, and blue). The first PPI cluster (Figure 4A (indicated in red) includes 30 proteins such as HLA-B, HLA-DQB1, HLA-DRB5, HLA-DRB6, HLA-S, HSPA1A, LY6G5BLY6G5C, LY6G6C, LY6G6D, MICB, ZBTB12, AIF1, LST1, MICA, etc.) was represented mainly by interactions of proteins associated with the innate/adaptive immune responses: Interferon-gamma-mediated signaling pathway (GO:0060333, p_FDR_ = 1.97 × 10^−5^), Antigen processing and presentation of peptide antigen (GO:0048002, p_FDR_ = 5.19 × 10^−5^), Regulation of immune system process (GO:0002682, p_FDR_ = 0.002), Immune response-activating cell surface receptor signaling pathway (GO:0002429, p_FDR_ = 0.009), Cytokine-mediated signaling pathway (GO:0019221, p_FDR_ = 0.02), etc. The second PPI cluster (Figure 4B (indicated in green) also includes 30 proteins such asHSPA1B, HSPA1A, C4A, C4B, CLIC1, BAG6, FLOT1, etc.) was characterized by interactions of heat shock proteins, complement systems proteins, etc., in which such pathways as Blood microparticle (GO:0072562, p_FDR_ = 0.002), Extracellular exosome (GO:0070062, p_FDR_ = 0.004), Misfolded protein binding (GO:0051787, p_FDR_ = 0.04), etc., are involved. The third PPI cluster (Figure 4C (indicated in blue) includes 15 proteins such as HIST1H2BC, HIST1H2AC, HIST1H4C, etc.) is distinguished by the interaction of various histone proteins based on such Reactome pathways as recognition and association of DNA glycosylase with sites containing an affected pyrimidine (HSA-110328, p_FDR_ = 0.005) and purine (HSA-110330, p_FDR_ = 0.005), Cleavage of the damaged pyrimidine (HSA-110329, p_FDR_ = 0.005) and purine (HSA-110331, p_FDR_ = 0.005), Meiotic synapsis (HSA-1221632, p_FDR_ = 0.005), Packaging Of Telomere Ends (HSA-171306, p_FDR_ = 0.005), Pre-NOTCH Transcription and Translation (HSA-1912408, p_FDR_ = 0.005), Formation of the beta-catenin:TCF transactivating complex (HSA-201722, p_FDR_ = 0.005), PRC2 methylates histones and DNA (HSA-212300, p_FDR_ = 0.005), Condensation of Prophase Chromosomes (HSA-2299718, p_FDR_ = 0.005), etc.

## 3. Discussion

In the present study associations have been replicated of the rs1799945 (C/G) *HFE* and intergenic interactions of seven GWAS-significant loci for AH/BP (rs1173771 (G/A) *AC026703.1*, rs1799945 (C/G) *HFE*, rs805303 (G/A) *BAG6*, rs932764 (A/G) *PLCE1*, rs4387287 (C/A) *OBFC1*, rs7302981 (G/A) *CERS5*, rs167479 (T/G) *RGL3*) with AH in the Caucasian population of Central Russia. AH risk SNPs (with more 80 proxy variants) are supposedly functionally efficient with respect to the 101 genes implicated in various immune system pathways. Three studied SNPs (rs8068318 (T/C) *TBX2*, rs633185 (C/G) *ARHGAP42*, rs2681472 (A/G) *ATP2B1*) did not confirm the association with AH.

Among the 10 GWAS AH/BP polymorphisms studied, we confirmed independent associations with AH for only one locus—rs1799945 (C/G) *HFE* gene (OR = 2.53 for genotype GG). There are quite numerous literature data (based on the results of GWAS) convincingly indicating the involvement of the SNP rs1799945 (C/G) *HFE* in the BP levels formation [29,30,31,32,33,34,35] and susceptibility to AH development [29,31].

In three GWAS, a high BP level and an increased risk of AH have been marked by the genetic variant G rs1799945 [29,31,34], and in three other GWAS, lower BP values (systolic/diastolic/mean/pulse blood pressure) were marked by a reference allele C of this polymorphism [30,31,32,33]. In our work, the risk influence on the AH development is exerted by the genotype GG rs1799945 (C/G) *HFE* (OR = 2.53), which fully corresponds to the above-mentioned literature GWAS data on the role of allelic variants of this locus (G-risky vs. C-protective) in the formation of BP and AH in various populations of the world.

Rather interesting results about the biological significance of the rs1799945 (C/G) *HFE* in humans of European ancestry, which can to some extent give a biomedical explanation of the relationship of this polymorphism with susceptibility to AH, were obtained in the study Gill et al. [36].

The authors, on the one hand, on the basis of GWAS data, showed a strong link between the locus rs1799945 (C/G) *HFE* and the human organism iron status (these were analyzed such serum parameters as levels of the iron, transferrin, ferritin, and saturation of transferrin), on the other hand, using a phenome-wide association study with mendelian randomization (MR-PheWAS analyses), established a substantial influence (causal effect) of the status of iron in organisms at risk of anemia and hypercholesterolemia development [36]. A direct link between hypercholesterolemia and a high AH risk (and in general, a high risk of morbidity/mortality associated with cardiovascular diseases) has been known for a long time and is currently beyond doubt (including in the countries of Eastern Europe) [2,37]. High levels of total cholesterol (TC), triglycerides (TG), low-density lipoprotein cholesterol (LDL-C), and low levels of high-density lipoprotein cholesterol (HDL-C) in AH patients in comparison with the control were also found in the studied sample (Table 3), which, firstly, confirms the significant role of hypercholesterolemia in AH development (risk factor); secondly, it gives reason to assume that one of the significant mechanisms underlying the involvement of rs1799945 (C/G) *HFE* in AH formation in the population studied by us may be iron status and hypercholesterolemia associated with it.

The data derived by us in silico also demonstrate a serious functional potential of the rs1799945 (C/G) *HFE* in the organism (it and 7 loci strongly linked to it have been functionally important for 15 genes, including replacement of amino acid Histidine by Aspartic acid in the 63 position of the HFE protein, epigenetic changes in 5 genes (*HIST1H2BC; HIST1H2AC; HIST1H1T; HIST1H4C; HFE*), eQTL/sQTL influences on 11/1 genes (*ALAS2; BTN2A3P; GUSBP2; HFE; HIST1H3E; RP11-457M11.5; SLC17A1; SLC17A3; TRIM38; U91328.19; ZNF322*/*HFE*)), which can also justify its involvement in AH susceptibility. For example, histone genes, such as *H2A, H3E, H1T, H2B, H4C,* etc., are functionally associated with the rs1799945 locus and are of paramount importance in the regulation of chromatin structure resulting in the modification of DNA “activity” (suppress/activate gene transcription): H2A variants hold the positions of entry/exit along the nucleosomal DNA wrap and thus control the “availability” of DNA; various fractions of H3 histone proteins and their modifications are known “markers” of functionally active DNA regions (promoters/enhancers); the H1T variant is a linker histone and “coordinates” chromatin packaging [38]. In another example, using the cDNA library of the heart of an early human embryo, a new zinc finger gene called *ZNF322* was isolated, which, as the authors showed, is expressed both at various embryonic stages (from 80 days to 24 weeks; thus, it is in numerous tissues of an adult [39]. The authors have shown that ZNF322 is a “transcription activator” (via MAPK signaling pathways) of reporter genes such as SRE, AP-1, which have been important for AH pathobiology [39].

Using the in silico approach widely utilized in modern genetic research, we established the multifarious pleiotropic functional actions (chromatin changes; eQTL/sQTL) of 7 AH-involved loci (and 81 linked SNPs) in relation to 101 genes. Furthermore, for a considerable number of genes (more 20 genes), these influences were registered by us in AH target organs such as arterial vessels (aorta, coronary artery, etc.) (*OBFC1, HLA-DRB5, HLA-DRB1, HLA-DRB6*, *LY6G5B*, *VWA7*, *C4A*, *HFE*, *ABHD16A*, *CYP21A1P*, *SMARCD1*, *LY6G5C*, *COX14*, *GPANK1*, *RP4-605O3.4*, *RP4-605O3.4*, *BAG6*, *ATF1*, *LSM2*, *STK19B*, *ATF6B*) and adult/fetal heart (*OBFC1*, *HLA-DRB1*, *HLA-DRB6*, *HLA-DRB5*, *CSNK2B*, *C4A*, *CERS5*, *CYP21A1P*, *HIST1H3E*, *DDAH2*, *RP4-605O3.4*, *STN1*, *LY6G5B*, *STK19B*, *LY6G5C*, *GPANK1*). The phenotypic functionality of the aforementioned genes may be responsible for the pathophysiology of AH. Thus, for instance, the *OBFC1* gene (also called *STN1 subunit of CST complex*) encodes one of the subunits of alpha accessory factor that stimulates the enzyme activity and thereby initiates DNA replication; as well, this protein is important for telomere-linked complexes and telomere regulation mechanisms (it provides telomere length homeostasis by inhibiting telomerase, recruits and activates the corresponding DNA polymerase, facilitates repair and initiates DNA replication, etc.) [40,41]. Said et al. detected the strongest association of genetically determined telomere length (including *OBFC1* gene) with cardiovascular disease and hypertension (based on Mendelian randomization data of UK Biobank 134,773 individuals) [42]. The strong effect of expression-involved *OBFC1* polymorphic variants (including eQTL in adventitial tissue of aorta) on cardiovascular disease risk has been shown in previous publication data [43]. Considering our in silico data, several genes of the body immune system (*HLA-DRB1*, *HLA-DRB6*, *HLA-DRB5*) and the extensive pathways related with them associated with the immune responses (MHC protein-involved reactions, immune system process regulation, antigen processing and presentation, etc.) are among the causal factors of hypertension. Pronounced changes in the immune system of AH patients (increased proinflammatory interleukins and other cytokines (tumor necrosis factors, chemokines, etc.) plasma levels, heat shock proteins overexpression, etc.), a substantial contribution of various immune-dependent processes to the development/aggravation of the disease (innate/adaptive immunity activation, changes of the immune reactivity, B- and T- cells pro- and anti- inflammatory responses, manifestations of autoantigenic reactions (due to heat shock proteins, isoketal-modified proteins, etc.), cascade of cytokine responses, etc.), and the need to correct the immune imbalance during hypertension therapy have been noted in numerous previous studies [44,45,46,47]. Rodriguez-Iturbe et al. considers the formation and exacerbation of hypertension as a step-by-step process of involving a variety of mechanisms of the immune system (episodic formation of danger-linked molecular substations and Toll-like receptor expression, activation of the innate immune system and the appearance of inflammatory reactions (infiltration, etc.) in target organs (kidney, vessels, etc.), involvement adaptive immunity reactions in the renal and vascular inflammation, the appearance of an imbalance in inflammatory/anti-inflammatory responses, etc.) and indicate the need to identify genetic factors/traits of hypertension related to the immune response [44]. Our work confirms the paramount importance of genetic factors (including certain AH-impact GWAS polymorphisms) associated with the multiple immune system pathways in AH formation in the Caucasian population of Central Russia.

It is extremely interesting that in a previously conducted genetic research aimed at finding associations of GWAS AH candidate genes with pre-eclampsia in a sample that included 452 pregnant women with pre-eclampsia and 498 pregnant women without pre-eclampsia (the same ten GWAS AH/BP risk loci panel was studied in the same Caucasian population of Central Russia), the significant role of rs1799945 (C/G) *HFE* (OR = 2.24) in the development of this pregnancy complication was detected [48]. Herewith, it is important to note the complete coincidence of the data obtained for AH (this study) and pre-eclampsia (obtained by us earlier [48]) in orientation (the risk value of the polymorphic variant G) and the associated strength of the polymorphism rs1799945 (C/G) *HFE* (OR indicators were approximately the same in value and amounted to OR = 2.53 for AH and OR = 2.24 for pre-eclampsia). These facts may indicate, on the one hand, the “reliability” of the identified associations obtained from completely different samples in the same ethno-territorial group of the Russian population and, on the other hand, the proven risk role of GWAS-impact rs1799945 (C/G) *HFE* in the development of hypertensive conditions (AH as an independent disease, and arterial hypertension is the main symptom of pre-eclampsia) in the Caucasian population of Central Russia, which opens up good prospects for further use of this polymorphism with a prognostic purpose in the practical medicine in this territory of Russia. Meanwhile, there is also an obvious need for additional evidence of the effectiveness of the rs1799945 (C/G) *HFE* polymorphism in other human diseases correlated with hypertensive manifestations (for example, kidney disease, metabolic syndrome, etc.) in the Caucasian population of Central Russia, which may be the subject of further research.

## 4. Materials and Methods

### 4.1. Study Subjects

When planning this work, the number of samples (patient/control) necessary for the study were determined using the Genetic Association Study (GAS) Power Calculator software (online source: http://csg.sph.umich.edu/abecasis/gas_power_calculator/ (accessed on 18 November 2022)) (a multiplicative model of the disease was considered). Taking into account the prevalence of AH among adults, according to the literature [1], on average, about 30–45% with the required research power of 80% and 5% error of the 1st kind (α = 0.05) to identify differences in the frequencies of polymorphic variants between patients and control at the level of OR = 1.23–1.30, the total sample number (patients and control) should be at least 1100 subjects with the prevalence of polymorphic genetic variants among the population ≥10%.

The sample of the present case-control study consisted of 1405 unrelated Caucasian participants (Russian origin; born in Central Russia [49,50]) including 939 AH patients and 466 AH free individuals. The studied subjects were recruited during the 2013–2016 period at the Cardiology Department of St. Joasaph Belgorod Regional Clinical Hospital. All procedures in this study were performed following the tenets of the Declaration of Helsinki. The study protocol was approved by the of the Human Investigation Committee (Ethics Committee) of Belgorod State University. All the participants were fully informed of the purpose and procedures, and written consent was obtained from each participant. The AH diagnosis was verified by certified cardiologists according to the WHO/ESC/ESH recommendation [1] (this was described in detail by us earlier [21]). AH was defined as SBP ≥ 140 mmHg and/or DBP ≥ 90 mmHg (office parameters) [1]. All AH patients had ≥1 year AH clinical anamnesis, and 81.79% received antiAH drugs. Control subjects were AH free (SBP < 140mmHg and DBP < 90mmHg), did not receive antiAH drugs and did not have pronounced metabolic (type 2 diabetes mellitus) and cardiovascular (coronary artery disease) disorders. Individuals with severe autoimmune/allergic/oncological/hematological disorder were excluded [51]. Data characterizing diet and lifestyle were obtained for all subjects. In accordance with WHO/FAO Expert recommendations, “low fruit/vegetable consumption” was considered as daily consumption of less than 400 g of fruits and vegetables, “low physical activity” was evaluated as average weekly physical activity of medium intensity (total at home and at work) less than 2.5 h, “high fatty foods consumption” was estimated to be the share of the average daily consumption of fatty foods from the total amount of food consumed ≥10%, and “high sodium consumption” was considered to be the daily consumption of salt in the volume of a teaspoon or more (≥5 g) [52].

Table 3 presents phenotypic characteristics of the AH and AH-free participants previously received by us [21]: AH patients had high indicators of BMI, blood glucose, TC, TG, LDL-C, smokers and low parameters of HDL-C vs. AH-free individuals (*p* < 0.001). These data gave us a reason to use the above indicators as confounders in genetic analysis (Model 1). Besides this, the percentage of individuals with low physical activity and preferring fatty foods was higher among AH patients (*p* < 0.001) (Table 3). These two AH-significant risk factors were additionally included as covariates in Model 2 (together with all covariates of Model 1).

### 4.2. Experimental Genetic Analysis (DNA Isolation; SNPs Selection; SNPs Genotyping)

Five milliliters of the venous blood was drawn from the ulnar vein and collected into tubes containing 100 μL of 10% EDTA [53]. High molecular weight genomic DNA was extracted from peripheral blood leukocytes, using the standard (phenol/chloroform) protocols [54].

For this study, we selected 10 loci taking into account special criteria such as (1) previously GWAS-linked with BP/AH in Caucasians; (2) possessed impact functionality (evaluated in silico by HaploReg programme [55]), and (3) had significant polymorphism (the frequency of the minor allele was at least 10% among Europeans (HaploReg data [55] and the data of previously conducted studies [48,56,57] were taken into account)). The ten common SNPs (rs167479 of *RGL3*, rs8068318 of *TBX2*, rs2681472 of *ATP2B1*, rs7302981 of *CERS5*, rs633185 of *ARHGAP42*, rs4387287 of *OBFC1*, rs932764 of *PLCE1*, rs805303 of *BAG6*, rs1799945 of *HFE*, rs1173771 of *AC026703.1*) were chosen for this study based on the above special criteria. All ten SNPs were BP-associated in Europeans, and all 10 SNPs were AH-linked: eight loci were AH-correlated in Europeans and two SNPs (rs2681472 *ATP2B*1; rs4387287 *OBFC1*) were AH-significant in the cohort subjects with European prevalence (more 85% out all participations) (Table S13). Nine polymorphic variants among studied 10 SNPs where AH/BP was associated in at least two GWAS (only the locus rs4387287 *OBFC1* was AH/BP-associated in one GWAS) (Table S13). All 10 loci selected for the study were found with a frequency of 14% or more among Europeans (Table S14) and were functional (Table S14).

Well known and widely used in genetic research, the TaqMan probe method of Polymerase Chain Reaction was used for genotyping DNA on the examined SNPs. The allele-detection process was performed on a CFX96 Real-Time System (Applied Biosystems, Foster City, CA, USA) to determine the allelic discrimination [58]. The genotyping procedure was carried out at the department of Medical Biological Disciplines (Laboratory of “Human Molecular Genetics”) of Belgorod State National Research University. We paid special attention to the quality control of genotyping: duplicate samples of DNA of participants (≈4–6% out all samples) (independent internal positive control) and samples containing the reaction mixture but not the DNA content (independent internal negative control) were interspersed throughout the plates used for genotype analysis for quality control purposes [59,60]. Importantly, the status of “AH/AH free” of the subjects and the positions of positive/negative independent internal controls on the plates were “hidden” for laboratory personnel during the entire procedure of experimental genetic research. The concordance rates for quality control samples (positive/negative internal controls) were 100% for all assays. An additional measure of quality control of genotyping was the assessment of the compliance of the observed distribution of genotypes with the expected parameters when fulfilling the Hardy–Weinberg law (we performed this procedure at the next stage of our study—statistical analysis of genetic data). The implementation of the Hardy–Weinberg law for the loci under consideration was an additional argument indicating the sufficient quality of the genotyping performed.

### 4.3. Statistical Analysis of Genetic Data

The samples included in the study (939 AH and 466 AH free) can identify differences in the frequencies of minor alleles of studied SNPs (with an estimated prevalence of the SNPs minor allele among Europeans of 14–46% (Table S14) (HaploReg data [55]) between the AH and AH free groups at OR = 1.25–1.37 (additive model), OR = 1.42–1.44 (dominant model), OR = 1.47–3.10 (recessive model) (calculations were performed in the Quanto program (http://hydra.usc.edu/gxe, 2006 (accessed on 18 November 2022)), at power = 80%; α = 0.05 for 2-sided test).

For each SNP, the Hardy–Weinberg equilibrium was assessed by Pearson’s goodness-of-fit χ2 statistical test [61,62]. The degrees of significance of differences in polymorphic variants (genotype/allele and genotype combinations frequencies) of studied genes (four inheritance models were considered for individual SNPs (allelic; recessive; additive; dominant) [63]; for the general SNP list, intergenic interactions were analyzed [64]) between AH and AH-free cohorts were calculated using gPLINK [65], MB-MDR [66,67], MDR [68,69] packages. The odds ratios (OR_genetic model_) and their 95% confidence intervals (_CIgenetic model_) were obtained by logistic regression [70,71] while adjusting for multiple comparisons (applied permutation testing [72]) and confounding factors (listed above for Model 1 and Model 2 in the “Study subjects” section according to the data in Table 3). Statistical significance was set at 5%, or p_perm_ (p_perm-interaction_) ≤ 0.05. For AH-associated SNP rs1799945 (C/G) *HFE* was calculated for statistical power with Quanto tool (http://hydra.usc.edu/gxe, 2006 (accessed on 18 November 2022)).

### 4.4. SNPs/Gene Predict Functionality/Functions

The well-known bioinformatics information (PolyPhen [73]; SIFT [74], HaploReg [55]; Blood eQTL browser [75]; GTExConsortium [76]; Gene Ontology [77]; GeneMANIA [78]; STRING [79]) was applied to examine in silico an association at the AH-involved loci/genes and high-linked SNPs (used parameter r^2^ equal or more 0.80 [80,81]) (according to the HaploReg database [55]) with functional prediction effects [82,83].

## 5. Conclusions

The GWAS-impact for AH/BP polymorphic locus rs1799945 of the *HFE* gene and intergenic interactions of *BAG6, CERS5*, *AC026703.1*, *HFE*, *PLCE1*, *OBFC1*, *RGL3* are associated with the risk of developing AH in the Caucasian population of Central Russia. Alongside this, three studied SNPs such as rs8068318 (T/C) *TBX2*, rs633185 (C/G) *ARHGAP42,* rs2681472 (A/G) *ATP2B1,* did not confirm the association with AH.

## Figures and Tables

**Table 1:** Associations of the studied gene polymorphisms with AH.

Gene (SNP, Major/Minor Alleles)	n	Allelic Model				Additive Model				Dominant Model				Recessive Model			
		OR	95% CI		*p*	OR	95% CI		*p*	OR	95% CI		*p*	OR	95% CI		*p*
			L95	U95			L95	U95			L95	U95			L95	U95	
**Model 1**																	
*AC026703.1* (rs1173771,G/A)	1317	0.90	0.77	1.06	0.216	0.89	0.69	1.14	0.354	0.75	0.51	1.10	0.140	1.03	0.65	1.62	0.905
*HFE* (rs1799945,C/G)	1373	0.94	0.77	1.15	0.550	1.04	0.78	1.40	0.781	0.90	0.63	1.28	0.559	**2.53**	**1.03**	**6.23**	**0.043**
*BAG6* (rs805303,G/A)	1349	0.97	0.82	1.14	0.683	0.87	0.68	1.11	0.264	0.95	0.67	1.34	0.750	0.65	0.40	1.04	0.075
*PLCE1* (rs932764,A/G)	1319	0.87	0.74	1.02	0.094	0.81	0.63	1.04	0.096	0.65	0.43	1.02	0.056	0.88	0.58	1.32	0.525
*OBFC1* (rs4387287,C/A)	1260	0.90	0.67	1.22	0.550	0.90	0.65	1.25	0.542	0.90	0.61	1.33	0.605	0.79	0.31	1.99	0.611
*ARHGAP42* (rs633185,C/G)	1377	1.02	0.85	1.22	0.813	1.09	0.84	1.42	0.525	1.08	0.76	1.52	0.673	1.25	0.68	2.32	0.472
*CERS5* (rs7302981,G/A)	1302	1.03	0.87	1.22	0.711	0.94	0.73	1.21	0.615	1.03	0.71	1.48	0.882	0.76	0.47	1.22	0.249
*ATP2B1* (rs2681472,A/G)	1329	1.04	0.82	1.31	0.762	1.17	0.82	1.67	0.384	1.17	0.79	1.74	0.437	1.51	0.41	5.50	0.532
*TBX2* (rs8068318,T/C)	1292	1.10	0.92	1.33	0.297	1.14	0.86	1.52	0.356	1.17	0.82	1.66	0.398	1.25	0.61	2.55	0.541
*RGL3* (rs167479,T/G)	1333	0.93	0.79	1.09	0.367	0.82	0.64	1.05	0.110	0.86	0.57	1.29	0.460	0.69	0.47	1.02	0.061
**Model 2**																	
*AC026703.1* (rs1173771,G/A)						0.89	0.69	1.15	0.374	0.76	0.51	1.11	0.153	1.03	0.65	1.64	0.893
*HFE* (rs1799945,C/G)						1.01	0.75	1.36	0.946	0.87	0.61	1.25	0.465	**2.48**	**1.02**	**6.07**	**0.045**
*BAG6* (rs805303,G/A)						0.89	0.69	1.14	0.343	0.98	0.69	1.39	0.906	0.65	0.40	1.06	0.085
*PLCE1* (rs932764,A/G)						0.81	0.63	1.04	0.093	0.64	0.42	1.02	0.054	0.88	0.58	1.33	0.535
*OBFC1* (rs4387287,C/A)						0.88	0.63	1.22	0.448	0.87	0.59	1.29	0.490	0.78	0.30	2.01	0.608
*ARHGAP42* (rs633185,C/G)						1.11	0.85	1.44	0.455	1.01	0.78	1.56	0.592	1.28	0.69	2.36	0.441
*CERS5* (rs7302981,G/A)						0.93	0.72	1.21	0.600	1.01	0.70	1.46	0.948	0.76	0.47	1.24	0.275
*ATP2B1* (rs2681472,A/G)						1.15	0.80	1.66	0.451	1.17	0.78	1.76	0.448	1.20	0.32	4.52	0.792
*TBX2* (rs8068318,T/C)						1.15	0.86	1.54	0.348	1.17	0.84	1.72	0.324	1.14	0.55	2.36	0.716
*RGL3* (rs167479,T/G)						0.80	0.63	1.03	0.078	0.81	0.54	1.22	0.320	0.68	0.46	1.01	0.057

Note: For Model 2, calculations of the allelic model were not performed because their results are identical to those of Model 1 (covariates are not used when calculating the allelic model). All results were obtained after adjustment for covariates. List covariates for Model 1: BMI, TC, TG, LDL-C, HDL-C, blood glucose, smokers. List covariates for Model 2: BMI, TC, TG, LDL-C, HDL-C, blood glucose, smokers, low physical activity, high fatty foods consumption. OR—odds ratio; 95% CI—95% confidence interval; p_perm_ values ≤ 0.05 are shown in bold.

**Table 2:** SNP × SNP interactions significantly associated with AH.

N	SNP × SNP Interaction Models	NH	*beta*H	WH	NL	*beta*L	WL	p_perm_
Two-order interaction models								
1	rs7302981 *CERS5* × rs805303 *BAG6*	2	0.372	8.02	3	−0.433	9.26	0.037
2	rs805303 *BAG6* × rs1173771 *AC026703.1*	1	1.318	7.99	2	−0.498	9.44	0.047
Three-order interaction models								
1	rs932764 *PLCE1* × rs7302981 *CERS5* × rs805303 *BAG6*	1	0.766	3.74	2	−0.799	22.25	0.001
2	rs932764 *PLCE1* × rs805303 *BAG6* × rs4387287 *OBFC1*	2	0.861	14.33	5	−0.668	20.70	0.006
3	rs7302981 *CERS5* × rs805303 *BAG6* × rs1173771 *AC026703.1*	3	0.575	11.10	3	−1.085	20.18	0.006
Four-order interaction models								
1	rs7302981 *CERS5* × rs805303 *BAG6* × rs1173771 *AC026703.1* × rs167479 *RGL3*	1	0.659	6.64	10	−1.089	47.36	<0.001
2	rs7302981 *CERS5* × rs1799945 *HFE* × rs805303 *BAG6* × rs167479 *RGL3*	2	0.682	10.03	9	−1.085	43.66	<0.001
3	rs932764 *PLCE1* × rs7302981 *CERS5* × rs805303 *BAG6* × rs167479 *RGL3*	3	0.895	11.66	6	−1.081	38.10	0.001

Note: The results were obtained using the MB-MDR method with adjustment for covariates (Model 1); NH—number of significant high risk genotypes in the interaction; *beta*H—regression coefficient for high risk exposition in the step2 analysis; WH—Wald statistic for high risk category; NL—number of significant low- risk genotypes in the interaction; *beta*L—regression coefficient for low risk exposition in the step2 analysis; WL—Wald statistic for low risk category; p_perm_—permutation *p* value for the interaction model (1.000 permutations).

**Table 3:** Phenotypic characteristics of the study participants.

Parameters	AH, Mean ± SD, % (n)	Controls, Mean ± SD, % (n)	*p*
N	939	466	
Gender (Male/ Female)	60.06/39.94 (564/375)	55.15/44.85 (257/209)	0.09
Age (years)	58.08 ± 8.91	57.82 ± 9.52	0.77
BMI (kg/m^2^)	30.78 ± 5.08	24.94 ± 3.14	<0.001
SBP (mmHg)	182.48 ± 28.26	122.58 ± 11.49	<0.001
DBP (mmHg)	105.84 ± 13.47	77.65 ± 6.93	<0.001
TC (mM)	5.71 ± 1.29	5.26 ± 1.04	<0.001
HDL-C (mM)	1.34 ± 0.42	1.52 ± 0.42	<0.001
LDL-C (mM)	3.78 ± 1.11	3.22 ± 0.74	<0.001
TG (mM)	1.92 ± 1.03	1.22 ± 0.71	<0.001
BG (mM)	5.92 ± 1.68	4.88 ± 0.95	<0.001
Smoking	38.33 (353)	19.76 (84)	<0.001
Alcohol abuse	5.79 (53)	3.12 (13)	0.051
Low physical activity	58.68 (551)	27.47 (128)	<0.001
Low fruit/vegetable consumption	11.39 (107)	8.15 (38)	0.074
High fatty foods consumption	24.71 (232)	10.30 (48)	<0.001
High sodium consumption	16.72 (157)	13.30 (62)	0.113

Note: Clinical characteristics of age, BMI, SBP, DBP, HDL-C, LDL-C, TG and TC are given as means ± SD and other values as number of individuals; BMI—body mass index; BG—blood glucose; SBP—systolic blood pressure; DBP—diastolic blood pressure; TC—total cholesterol; HDL-C—high-density lipoprotein cholesterol; TG—triglycerides; LDL-C—low-density lipoprotein cholesterol.

## Data Availability

The data generated in the present study are available from the corresponding author upon reasonable request.

## Supplementary Materials

The following supporting information can be downloaded at: https://www.mdpi.com/article/10.3390/ijms24098309/s1. References [84,85,86,87,88,89,90,91,92,93,94,95,96,97,98,99,100] are cited in Supplementary Materials.
