# Supplementary material for: Risk Effects of rs1799945 Polymorphism of the HFE Gene and Intergenic Interactions of GWAS-Significant Loci for Arterial Hypertension in the Caucasian Population of Central Russia
Source: Int J Mol Sci. 2023 May 5;24(9):8309. doi: 10.3390/ijms24098309 (PMC10179076; doi:10.3390/ijms24098309)
Supplement: Supplementary file 1 [file ijms-24-08309-s001.zip › Suppl Table S1.pdf]

Supplementary Table S1. The allele and genotype frequencies of the studied SNPs in the AH and control groups

Gene (SNP, major/minor alleles)	Minor allele frequency,%	Genotype distribution *	H_o	H_e	P_{HWE}
AH patients (n=939)					
AC026703.1 (rs1173771,G/A)	42.51	155/433/286	0.495	0.489	0.729
HFE (rs1799945,C/G)	19.34	42/270/603	0.295	0.312	0.111
BAG6 (rs805303,G/A)	33.84	114/379/404	0.423	0.448	0.101
PLCE1 (rs932764,A/G)	48.79	206/438/227	0.503	0.500	0.892
OBFC1 (rs4387287,C/A)	19.07	39/242/558	0.288	0.309	0.106
ARHGAP42 (rs633185,C/G)	27.01	77/343/500	0.373	0.394	0.112
CERS5 (rs7302981,G/A)	39.77	136/416/313	0.481	0.479	0.944
ATP2B1 (rs2681472,A/G)	14.37	20/214/650	0.242	0.246	0.587
TBX2 (rs8068318,T/C)	26.85	71/316/466	0.371	0.393	0.098
RGL3 (rs167479,T/G)	49.38	211/453/222	0.511	0.500	0.545
Control group (n=466)					
AC026703.1 (rs1173771,G/A)	45.03	85/229/129	0.517	0.495	0.388
HFE (rs1799945,C/G)	20.31	20/146/292	0.319	0.324	0.773
BAG6 (rs805303,G/A)	34.62	60/193/199	0.427	0.453	0.253
PLCE1 (rs932764,A/G)	52.23	125/218/105	0.487	0.499	0.636
OBFC1 (rs4387287,C/A)	20.67	18/138/265	0.328	0.328	1.000
ARHGAP42 (rs633185,C/G)	26.59	34/175/248	0.383	0.390	0.719
CERS5 (rs7302981,G/A)	39.02	69/203/165	0.465	0.476	0.616
ATP2B1 (rs2681472,A/G)	13.93	10/104/331	0.234	0.240	0.555
TBX2 (rs8068318,T/C)	24.94	32/155/252	0.353	0.374	0.251
RGL3 (rs167479,T/G)	51.23	123/212/112	0.474	0.500	0.298

Note: * minor allele homozygotes/heterozygotes/major allele homozygotes
