# Supplementary material for: Risk Effects of rs1799945 Polymorphism of the HFE Gene and Intergenic Interactions of GWAS-Significant Loci for Arterial Hypertension in the Caucasian Population of Central Russia
Source: Int J Mol Sci. 2023 May 5;24(9):8309. doi: 10.3390/ijms24098309 (PMC10179076; doi:10.3390/ijms24098309)
Supplement: Supplementary file 1 [file ijms-24-08309-s001.zip › Suppl Table S13.pdf]

Supplementary Table S13. The GWAS data about associations of the studied candidate genes polymorphisms with blood pressure/hypertension, some cardiovascular diseases and anthropometric characteristics

SNP of gene (position (hg38))	Phenotype	Association (significance) (affected allele)	Reference
rs1173771 *AC026703.1* (5: 32814922)	SBP	β = 0.50 ($p = 1.8×10^{-16}$) (G)	International Consortium et al., 2011 [29]
	DBP	β = 0.26 ($p = 9.1×10^{-12}$) (G)	
	Hypertension	β = 0.06 ($p = 3.2×10^{-10}$) (G)	
	MAP	β = 0.28 ($p = 3.51×10^{-9}$) (G)	Wain L. V., et al., 2011 [98]
	PP	β = 0.28 ($p = 4.56×10^{-9}$) (G)	
	MAP	β = −0.35 ($p = 3.19×10^{-8}$) (A)	Kato N., et al., 2015 [89]
	Hip circumference (sex-combined)	β = 0.03 ($p = 6.13×10^{-13}$) (A)	Shungin D., et al., 2015 [95]
	Hip circumference (men)	β = 0.03 ($p = 6.19×10^{-8}$) (A)	
	SBP	β = −0.43 ($p = 2.0×10^{-28}$) (A)	Hoffmann T. J. et al., 2017 [33]
	PP	β = −0.25 ($p = 1.0×10^{-21}$) (A)	
	DBP	β = −0.19 ($p = 8.0×10^{-16}$) (A)	
	Height	β = 0.04 ($p = 9.0×10^{-8}$) (A)	Tachmazidou I., et al., 2017 [96]
	SBP	β = 0.51($p = 3.20×10^{-12}$) (G)	Wain L. V., et al., 2017 [34]
	PP	β = 0.28 ($p = 2.36×10^{-9}$) (G)	
	SBP (smoking interaction) (European)	$p = 1.74×10^{-38}$	Sung Y. J., et al., 2018 [35]
	DBP (smoking interaction) (European)	$p = 3.62×10^{-22}$	
	SBP (smoking interaction) (trans-ethnic data)	$p = 8.25×10^{-44}$	
	DBP (smoking interaction) (trans-ethnic data)	$p = 2.75×10^{-28}$	
	SBP	β = −0.42 ($p = 4.19×10^{-8}$) (A)	Takeuchi F., et al., 2018 [97]
	MAP	β = −0.29 ($p = 5.39×10^{-8}$) (A)	
rs1799945 *HFE* (6: 26090951)	DBP	β = 0.46 ($p = 1.5×10^{-15}$) (G)	International Consortium et al., 2011 [29]
	SBP	β = 0.62 ($p = 7.7×10^{-12}$) (G)	
	Hypertension	β = 0.10 ($p = 1.8×10^{-10}$) (G)	
	DBP	β = −0.43 ($p = 3.1 ×10^{-16}$) (C)	Ehret G. B. et al., 2016 [32]
	SBP	β = −0.60 ($p = 3.28 ×10^{-12}$) (C)	
	DBP	β = −0.29 ($p = 1.0×10^{-18}$) (C)	Hoffmann T. J. et al., 2017 [33]
	SBP	β = −0.36 ($p = 4.0×10^{-11}$) (C)	

	DBP	β =0.52 (p = 9.2×10^{-18}) (G)	Liu C. et al., 2016 [31]
	MAP	β = 0.58 (p = 1.0×10^{-17}) (G)	
	SBP	β = 0.70 (p = 6.2×10^{-13}) (G)	
	Hypertension	Z score = 0.039 (p = 3.7×10^{-9}) (G)	
	DBP (trans-ethnic data)	β = −0.03 (p = 1.26×10^{-19}) (C)	Surendran P. et al., 2016 [30]
	DBP (European)	β = −0.03 (p = 9.82×10^{-10}) (C)	
	DBP	β = 0.47 (p = 8.87×10^{-14}) (G)	Wain L. V. et al., 2017 [34]
	SBP	β = 0.63 (p = 7.63×10^{-10}) (G)	
	DBP (smoking interaction) (European)	p = 1.51×10^{-31}	Sung Y. J. et al., 2018 [35]
	SBP (smoking interaction) (European)	p = 8.46×10^{-17}	
	DBP (smoking interaction) (trans-ethnic data)	p = 3.77×10^{-33}	
	SBP (smoking interaction) (trans-ethnic data)	p = 7.43×10^{-19}	
rs805303 *BAG6/ BAT2-BAT5* (6: 31648589)	SBP	β = 0.37 (p = 1.5×10^{-11}) (G)	International Consortium et al., 2011 [29]
	DBP	β = 0.23 (p = 3.0×10^{-11}) (G)	
	Hypertension	β = 0.05 (p = 1.1×10^{-10}) (G)	
	SBP (European)	β = −0.02 (p = 3.02×10^{-6}) (A)	Surendran P. et al., 2016 [30]
rs932764 *PLCE1* (10: 94136183)	SBP	β = 0.48 (p = 7.1×10^{-16}) (G)	International Consortium et al., 2011 [29]
	Hypertension	β = 0.06 (p = 9.4×10^{-9}) (G)	
	SBP	β = −0.50 (p = 6.88×10^{-17}) (A)	Ehret G. B., et al., 2016 [32]
	DBP	β = −0.22 (p = 6.28×10^{-10}) (A)	
	SBP	β = −0.30 (p = 1.0×10^{-14}) (A)	Hoffmann T. J. et al., 2016 [33]
	PP	β = −0.16 (p = 3.0×10^{-10}) (A)	
	PP	β = 0.26 (p = 5.67×10^{-8}) (G)	Wain L. V., et al., 2017 [34]
rs4387287 *OBFC1* (10:103918139)	DBP (European)	β= 0.22 (p=5.55 x 10^{-8}) (A)	Surendran P. et al., 2016 [30]
	SBP (European)	β= 0.34 (p=2.21 x 10^{-7}) (A)	
	DBP (trans-ethnic data)	β= 0.22 (p=4.21 x 10^{-10}) (A)	
	SBP (trans-ethnic data)	β= 0.36 (p=9.12 x 10^{-10}) (A)	
	Hypertension (trans-ethnic data)	Z score = 5.58 (p = 2.37 x 10^{-8}) (A)	
rs633185 *ARHGAP42* (11:100722807)	SBP	β = −0.57(p = 1.2×10^{-17}) (G)	International Consortium et al., 2011 [29]
	DBP	β = −0.33(p = 2.0×10^{-15}) (G)	
	Hypertension	β = −0.07(p = 6.4×10^{-11}) (G)	

	SBP	β = 0.52 (p = 6.97×10^{-15}) (C)	Ehret G. B., et al., 2016 [32]
	DBP	β = 0.29 (p = 2.38×10^{-12}) (C)	
	SBP	β = −0.49 (p = 1.0×10^{-31}) (G)	Hoffmann T. J. et al., 2016 [33]
	DBP	β = −0.27 (p = 1.0×10^{-26}) (G)	
	PP	β = −0.22 (p = 1.0×10^{-15}) (G)	
	SBP	β = 0.52 (p = 8.43×10^{-11}) (C)	Wain L. V., et al., 2017 [34]
	DBP	β = 0.27 (p = 2.33×10^{-8}) (C)	
	SBP x alcohol consumption interaction	β = 0.54 (p = 2.24×10^{-29}) (C)	Feitosa M. F. et al., 2018 [84]
	MAP x alcohol consumption interaction	β = 0.22 (p = 2×10^{-12}) (C)	
	MAP	β = 0.39 (p = 2.56×10^{-13}) (C)	Takeuchi F., et al., 2018 [97]
	SBP	β = 0.51 (p = 7.16×10^{-12}) (C)	
	DBP	β = 0.33 (p = 8.93×10^{-12}) (C)	
	Hypertension	β = 0.08 (p = 5.13×10^{-10}) (C)	
	Coronary artery disease	(p = 8.81×10^{-9}) (C)	Zhou W., et al., 2018 [99]
	High blood pressure and chronic obstructive pulmonary disease	(p = 1.18×$10^{-47)}$	Zhu Z., et al., 2019 [100]
	SBP (smoking interaction) (European)	p = 8.44×10^{-30}	Sung Y. J., et al., 2018 [35]
	DBP (smoking interaction) (European)	p = 2.68×10^{-30}	
	SBP (smoking interaction) (trans-ethnic data)	p = 1.80×10^{-40}	
	DBP (smoking interaction) (trans-ethnic data)	p = 1.18×10^{-40}	
	MAP	β = 0.03 (p = 3.05×10^{-50}) (C)	Sakaue S. et al., 2021 [94]
rs7302981 *CERS5/AC074032.1* (12:50144032)	Hypertension	Z score = 6.23 (p= 4.8 x 10^{-10}) (A)	Liu C. et al., 2016 [31]
	SBP	β = 0.37 (p=9.4 x 10^{-15}) (A)	
	DBP	β = 0.25 (p = 9.4 x 10^{-19}) (A)	
	DBP (European)	β = 0.25 (p = 1.38 x 10^{-17}) (A)	Surendran P. et al., 2016 [30]
	SBP (European)	β = 0.34 (p = 6.06 x 10^{-13}) (A)	
	Hypertension (European)	Z score = 6.07 (p = 1.28 x 10^{-9}) (A)	
	DBP (trans-ethnic data)	β = 0.25 (p = 2.60 x 10^{-19}) (A)	
	SBP (trans-ethnic data)	β = 0.35 (p = 9.94 x 10^{-19}) (A)	
	Hypertension (trans-ethnic data)	Z score = 6.17 (p = 6.82 x 10^{-10}) (A)	
rs2681472 *ATP2B1* (12: 89615182)	DBP	β = 0.50 (p = 1.47×10^{-9}) (A)	Levy D. et al., 2009 [91]
	Hypertension	β = 0.15 (p = 1.75×10^{-11}) (A)	
	Coronary artery disease	OR = 1.08 (p = 6.17×10^{-11}) (G)	Nikpay M., et al., 2015 [93]

	Myocardial infarction	OR = 1.08 (p = 6.03×10^{-9}) (G)	
	MAP	β = −0.59 (p = 1.1×10^{-21}) (G)	Liu C. et al., 2016 [31]
	SBP	β = −0.85 (p = 1.3×10^{-21}) (G)	
	DBP	β = −0.47 (p = 3.7×10^{-17}) (G)	
	Hypertension	β = −0.033 (p = 3.5×10^{-8}) (G)	
	Coronary artery disease	OR = 1.07 (p = 1×10^{-21}) (G)	Nelson C. P., et al., 2017 [92]
	SBP	β = 0.72 (p = 1.06×10^{-20}) (A)	Takeuchi F., et al., 2018 [97]
	DBP	β = 0.33 (p = 3.77×10^{-11}) (A)	
	MAP	β = 0.46 (p = 5.05×10^{-17}) (A)	
	PP	β = 0.40 (p = 5.49×10^{-14}) (A)	
	Hypertension	β = 0.07(p = 1.41×10^{-6}) (A)	
	Coronary artery disease (trans-ethnic data)	β = 0.060 (p= 6.8 x 10^{-25}) (G)	Koyama S., et al., 2020 [90]
	Coronary artery disease (Japanese)	β = 0.068 (p= 2.6 x 10^{-11}) (G)	
	Myocardial infarction	OR = 1.07 (p = 1.3×10^{-12}) (G)	Hartiala J. A., et al., 2020 [87]
	Myocardial infarction	β = 0.071 (p= 1.17 x 10^{-11}) (G)	Sakaue S. et al., 2021 [94]
rs8068318 *TBX2/ TBX2-AS1* (17:61406405)	MAP	β = −0.28 (p = 2.0×10^{-8}) (C)	Liu C. et al., 2016 [31]
	SBP	β = −0.42 (p = 3.9×10^{-17}) (C)	
	Hypertension	Z score = −6.96 (p = 3.0 x 10^{-12}) (C)	
	DBP	β = −0.26 (p = 3.0×10^{-18}) (C)	
	SBP (European)	β = 0.42 (p= 1.3 x 10^{-15}) (T)	Surendran P. et al., 2016 [30]
	DBP (European)	β = 0.26 (p= 1.95 x 10^{-16}) (T)	
	Hypertension (European)	Z score = 6.97 (p = 3.21 x 10^{-12}) (T)	
	DBP (trans-ethnic data)	β = 0.25 (p= 2.75 x 10^{-18}) (T)	
	SBP (trans-ethnic data)	β = 0.41 (p= 2.3 x 10^{-17}) (T)	
	Hypertension (trans-ethnic data)	Z score = 6.96 (p = 3.43 x 10^{-12}) (T)	
rs167479 *RGL3* (19:11416089)	SBP	β = 0.41 (p = 1.6 x 10^{-21}) (G)	Hoffmann T. J. et al., 2017 [33]
	DBP	β = 0.25 (p = 4.3 x 10^{-22}) (G)	
	PP	β = 0.18 (p = 3.2 x 10^{-8}) (G)	
	MAP	β = −0.30 (p = 7.3 x 10^{-11}) (T)	Liu C. et al., 2016 [31]
	Hypertension	Z score = −7.72 (p = 1.2 x 10^{-14}) (T)	
	SBP	β = −0.45 (p = 1.0 x 10^{-23}) (T)	
	DBP	β = −0.30 (p = 4.2×10^{-28}) (T)	
	DBP (European)	β= −0.33 (p = 1.99 x 10^{-31}) (T)	Surendran P. et al., 2016 [30]
	SBP (European)	β= −0.50 (p =1.49 x 10^{-26}) (T)	
	Hypertension (European)	Z score = −7.86 (p = 4.01 x 10^{-15}) (T)	

	DBP (trans-ethnic data)	β= −0.31 (p = 2.76 x 10^{-32}) (T)	
	SBP (trans-ethnic data)	β= −0.47 (p = 8.64 x 10^{-27}) (T)	
	Hypertension (trans-ethnic data)	Z score = −7.88 (p = 3.37 x 10^{-15}) (T)	
	SBP	β = −0.41 (p = 4.32 x 10^{-36}) (T)	Giri A. et el. 2018 [86]
	Hypertension	β = 0.05 (p = 8.0 x 10^{-16}) (G)	German C. A., 2019 [85]
	Hypertension	OR = 0.84 – 0.92 (p = 2.37 x 10^{-8}) (T)	Jeong H. et al., 2020 [88]
	SBP	β = −0.027 (p = 3.24 x 10^{-46}) (T)	Sakaue S. et al., 2021 [94]
	DBP	β = −0.026 (p = 2.24 x 10^{-48}) (T)	
	PP	β = −0.016 (p = 7.72 x 10^{-18}) (T)	
	MAP	β = −0.028 (p = 3.64 x 10^{-48}) (T)	

Notes: SBP - systolic blood pressure; DBP - diastolic blood pressure; MAP - mean arterial pressure; PP - pulse blood pressure; z-score - standard scores, β – effect, OR – odds ratio, p – significance level.
