# Supplementary material for: Risk Effects of rs1799945 Polymorphism of the HFE Gene and Intergenic Interactions of GWAS-Significant Loci for Arterial Hypertension in the Caucasian Population of Central Russia
Source: Int J Mol Sci. 2023 May 5;24(9):8309. doi: 10.3390/ijms24098309 (PMC10179076; doi:10.3390/ijms24098309)
Supplement: Supplementary file 1 [file ijms-24-08309-s001.zip › Suppl Table S2.pdf]

Supplementary Table S2. Genotype combinations associated with AH *.

Model	N	Genotype combinations	*beta*	P	Risk, High/Low
Two-order interaction models					
1	1	rs7302981 GA *CERS5* × rs805303 GG *BAG6*	0.71	0.014	H
2	2	rs805303 AA *BAG6* × rs1173771 GG *AC026703.1*	−0.42	0.050	L
	3	rs805303 GG *BAG6* × rs1173771 GA *AC026703.1*	−0.48	0.026	L
	4	rs805303 GG *BAG6* × rs1173771 AA *AC026703.1*	1.31	0.005	H
Three-order interaction models					
1	1	rs932764 GG *PLCE1* × rs7302981 GA *CERS5* × rs805303 GA *BAG6*	−0.88	0.0007	L
	2	rs932764 AA *PLCE1* × rs7302981 AA *CERS5* × rs805303 AA *BAG6*	0.76	0.050	H
	3	rs932764 AG *PLCE1* × rs7302981 AA *CERS5* × rs805303 AA *BAG6*	−0.65	0.002	L
2	4	rs932764 GG *PLCE1* × rs805303 AA *BAG6* × rs4387287 CC *OBFC1*	−1.82	0.034	L
	5	rs932764 AA *PLCE1* × rs805303 AA *BAG6* × rs4387287 CA *OBFC1*	−0.78	0.016	L
	6	rs932764 GG *PLCE1* × rs805303 GA *BAG6* × rs4387287 AA *OBFC1*	−0.46	0.049	L
	7	rs932764 AA *PLCE1* × rs805303 GA *BAG6* × rs4387287 AA *OBFC1*	0.84	0.001	H
3	9	rs7302981 AA *CERS5* × rs805303 AA *BAG6* × rs1173771 GG *AC026703.1*	−0.99	0.013	L
	10	rs7302981 GG *CERS5* × rs805303 GG *BAG6* × rs1173771 GA *AC026703.1*	−1.09	0.021	L
	11	rs7302981 AA *CERS5* × rs805303 GG *BAG6* × rs1173771 GA *AC026703.1*	−1.04	0.005	L
	12	rs7302981 GA *CERS5* × rs805303 AA *BAG6* × rs1173771 GA *AC026703.1*	0.35	0.050	H
	13	rs7302981 GA *CERS5* × rs805303 GG *BAG6* × rs1173771 AA *AC026703.1*	2.38	0.022	H
	14	rs7302981 AA *CERS5* × rs805303 GG *BAG6* × rs1173771 AA *AC026703.1*	2.34	0.034	H
Four-order interaction models					

1	1	rs7302981 AA *CERS5* × rs805303 AA *BAG6* × rs1173771 GG *AC026703.1* × rs167479 TG *RGL3*	−1.76	0.007	L
	2	rs7302981 AA *CERS5* × rs805303 GG *BAG6* × rs1173771 GA *AC026703.1* × rs167479 TG *RGL3*	−1.19	0.021	L
	3	rs7302981 GA *CERS5* × rs805303 AA *BAG6* × rs1173771 GA *AC026703.1* × rs167479 TG *RGL3*	0.66	0.010	H
	4	rs7302981 GA *CERS5* × rs805303 GA *BAG6* × rs1173771 AA *AC026703.1* × rs167479 TG *RGL3*	−0.63	0.019	L
	5	rs7302981 GG *CERS5* × rs805303 AA *BAG6* × rs1173771 GG *AC026703.1* × rs167479 GG *RGL3*	−2.70	0.021	L
	6	rs7302981 GA *CERS5* × rs805303 AA *BAG6* × rs1173771 GG *AC026703.1* × rs167479 GG *RGL3*	−1.14	0.019	L
2	7	rs7302981 AA *CERS5* × rs1799945 CC *HFE* × rs805303 GG *BAG6* × rs167479 TG *RGL3*	−0.97	0.047	L
	8	rs7302981 GA *CERS5* × rs1799945 CG *HFE* × rs805303 GA *BAG6* × rs167479 TG *RGL3*	−0.94	0.004	L
	9	rs7302981 AA *CERS5* × rs1799945 CG *HFE* × rs805303 GA *BAG6* × rs167479 TG *RGL3*	1.09	0.045	H
	10	rs7302981 GA *CERS5* × rs1799945 CC *HFE* × rs805303 AA *BAG6* × rs167479 TG *RGL3*	0.57	0.015	H
	11	rs7302981 GG *CERS5* × rs1799945 CC *HFE* × rs805303 GG *BAG6* × rs167479 GG *RGL3*	−2.15	0.050	L
	12	rs7302981 GG *CERS5* × rs1799945 CC *HFE* × rs805303 AA *BAG6* × rs167479 GG *RGL3*	−1.09	0.032	L
	13	rs7302981 GA *CERS5* × rs1799945 CG *HFE* × rs805303 AA *BAG6* × rs167479 GG *RGL3*	−1.28	0.008	L
3	14	rs932764 GG *PLCE1* × rs7302981 GA *CERS5* × rs805303 GG *BAG6* × rs167479 TG *RGL3*	1.31	0.046	H
	15	rs932764 GG *PLCE1* × rs7302981 GA *CERS5* × rs805303 GA *BAG6* × rs167479 TG *RGL3*	−1.23	0.0002	L
	16	rs932764 GG *PLCE1* × rs7302981 GA *CERS5* × rs805303 AA *BAG6* × rs167479 TG *RGL3*	0.85	0.050	H
	17	rs932764 AG *PLCE1* × rs7302981 AA *CERS5* × rs805303 AA *BAG6* × rs167479 TG *RGL3*	−0.89	0.003	L
	18	rs932764 AA *PLCE1* × rs7302981 GG *CERS5* × rs805303 AA *BAG6* × rs167479 GG *RGL3*	−1.16	0.043	L

* Genotype combinations are derived from the interaction models obtained by the MB-MDR method and described in Tables 3
